# Correction: Thyrotropin aggravates atherosclerosis by promoting macrophage inflammation in plaques

**DOI:** 10.1084/jem.2018147312212021c

**Published:** 2022-01-07

**Authors:** Chongbo Yang, Ming Lu, Wenbin Chen, Zhao He, Xu Hou, Mei Feng, Hongjia Zhang, Tao Bo, Xiaoming Zhou, Yong Yu, Haiqing Zhang, Meng Zhao, Laicheng Wang, Chunxiao Yu, Ling Gao, Wenjian Jiang, Qunye Zhang, Jiajun Zhao

Vol. 216, No. 5 | 10.1084/jem.20181473 | April 2, 2019

The authors regret that in their original paper, the *Tshr* siRNA sequence listed in the TSHR siRNA interference section of the Materials and methods was incorrect. The correct sequence is sense, 5′-ACCAGAAGCUUAACCUAUA-3′; anti-sense, 5′-GUACAACAAUGGAUUUACU-3′.

The error appears in print and in PDFs downloaded before December 20, 2021.

